# Amputation of the Right Arm at the Shoulder Joint

**Published:** 1843-06

**Authors:** T. A. Tellkampf

**Affiliations:** Cincinnati, Ohio


Art. II.—*Amputation of the Right Arm at the Shoulder Joint.* By T. A. Tellkampf, M. D., of Cincinnati, Ohio.

*Feb. 5th*, 1842. Anthony Newhouse, aged twenty-five, and of healthy and vigorous constitution, a workman in Mr. W's. steam-brewery near this city, got entangled in the connecting cogs of the large transverse wheels of the machinery of the establishment, was drawn in between them, and pulled through to the other side, where he was soon after found. The bones of the right hand were almost entirely crushed; the muscles of the forearm were torn and lacerated, and bore the marks of the cogs; the arm was broken and crushed in its central portion, the splinters of bone projecting here and there—forming almost an indistinguishable mass of bone, muscular substance, muscles, nerves, bloodvessels, &c. The nerves and vessels were to some extent isolated. The upper and lower extremities of the humerus were both split, the fissure extending into the joint, as was ascertained by motion. Besides these, the patient had received other and severe injuries. The skin and muscles along the right side down to the pelvis (upon the edge of which the wheels had apparently rolled off) bore the marks of the cogs; three ribs were fractured; and there were wounds from four to eight inches long in the abdominal parietes, some of them penetrating to the peritoneum. The patient was removed to a house adjoining the brewery, where on my arrival, I found him, faint from pain, not from hemorrhage of which there had been little. As there was reason to apprehend tetanus from the increasing sensibility of the patient, with the assistance of Dr. Emmerth, who was with the patient when I arrived, I prepared for operating on the arm immediately, amputation at the joint being evidently the only remedy that could be adopted.

The patient was put in the proper position and some of his fellow workmen directed to hold him. Dr. E. compressing the subclavian artery, I drew the arm a little outwards from the body, and passing the knife over the shoulder, I cut first downwards from the top of the shoulder on its upper and outer surface to about the point of insertion of the latissimus dorsi and teres major muscles; thence returning with it in the same cut to about two finger's breadth below the acromion, I passed downwards and inwards on the arm towards the point of insertion above named, and to within about two finger's breadth of it. With this nearly circular cut, the skin and all the muscles of the arm at the shoulder were severed and the socket laid bare; passing back in the wound, the socket was fully opened. Raising the arm up out of its socket and passing the knife over the head of the humerus, the remaining portion of the ligaments and the muscles underneath were cut, and then nothing remained but to sever the part of the skin left uncut between the ends of the first named nearly circular incision, which I did from without. I have followed in this the method of Langenbeck, who first introduced and applied it in Germany, and gave the operation the name of extirpation of the humerus. The time required is about half a minute. The wound that is left forms a perpendicular line.

The arteries and veins being secured, after a little I dressed and bandaged the arm, using sutures secured by strips of adhesive plaster. It is not necessary to give details of the subsequent treatment. The patient was placed in such a position as to keep the fractured ribs in apposition. Some tendency to cough was manifested; he complained of great pain in the right side of the chest and abdomen, and in the region of the kidneys particularly.

The following day (*Feb. 6th*) the wound was a little swollen, not painful; slight cough; the pains in the right side of the breast and abdomen increased.

*Feb. 7th.* The face flushed, pulse full and soft; tongue coated; no pains in the breast; breathing freer; no blood was raised with the phlegm from the cough; severe pains still in the right side of the abdomen, none in the left; bowels loose; urine brown, clear and without sediment.

*Feb. 9th.* The wound of the arm was healed about two-thirds down from the shoulder; moderate discharge of pus from the lower part of the wound. I found one of the wounds in the side discharging serum and pus from near the os sacrum, and the probe could there be introduced about four finger's breadth in the direction of the kidney; urine still clear and without sediment. Suppuration under the contused skin of the abdomen very copious; the most contused parts commenced mortifying, to which warm aromatic poultices were applied from the beginning.

*Feb. 14th.* Copious discharge of serum from the above named fistula: a seton applied, and after two or three days no serum flowed out; I opened this and several other fistulous canals with the knife.

*Feb. 16th.* The patient free from pain, and the suppuration under the skin of the abdomen very free, but favorable.

The first ligature of the shoulder was removed on the 18*th*, (thirteenth day from the injury). The patient improved gradually and equably. Upon the sloughing off of the skin of the abdomen, I had it dressed twice daily with lint. The only drawback to his recovery was his endeavoring (March 10th) to heal up the the suppurating surface on the abdomen faster than I had intended, by using of his own accord a solution of nitrate of silver I had formerly used on the other parts, and the favorable effect of which he had noticed. This checked the suppuration, brought on pains in the abdomen, and caused a swelling of the left leg. By reproducing the secretion, these symptoms passed off and the patient recovered rapidly. A generous, nourishing diet was directed for him, to keep up his strength whilst suppuration lasted. The wound of the arm was healed about the 5th of March. In the beginning of April, when the wounds of the abdomen were almost healed up, I applied two setons on the breast. The latter wound left a smooth surface, no folding of the skin so as to inconvenience him at all in walking or otherwise. He has regained his usual health.

March, 1843.

